# CASZ1b, the Short Isoform of CASZ1 Gene, Coexpresses with CASZ1a during Neurogenesis and Suppresses Neuroblastoma Cell Growth

**DOI:** 10.1371/journal.pone.0018557

**Published:** 2011-04-07

**Authors:** Zhihui Liu, Arlene Naranjo, Carol J. Thiele

**Affiliations:** 1 Pediatric Oncology Branch, Center for Cancer Research, National Cancer Institute, Bethesda, Maryland, United States of America; 2 Children's Oncology Group Statistics and Data Center, University of Florida, Gainesville, Florida, United States of America; Instituto Nacional de Câncer, Brazil

## Abstract

In *Drosophila,* the CASZ1 (*castor*) gene encodes a zinc finger transcription factor and is a neural fate-determination gene. In mammals, the CASZ1 gene encodes two major isoforms, CASZ1a with 11 zinc fingers and CASZ1b with 5 zinc fingers. CASZ1b is more evolutionally conserved since it is the only homologue found in *drosophila* and *Xenopus*. Our previous study showed that full length CASZ1 (CASZ1a) functions to suppress growth in neuroblastoma tumor. However, the function of CASZ1b isoform in mammals is unknown. In this study, realtime PCR analyses indicate that mouse CASZ1b (mCASZ1b) is dynamically expressed during neurogenesis. CASZ1b and CASZ1a co-exist in all the neuronal tissues but exhibit distinct expression patterns spatially and temporally during brain development. CASZ1b and CASZ1a expression is coordinately upregulated by the differentiation agent Retinoic Acid, as well as agents that modify the epigenome in neural crest derived neuroblastoma cell lines. In contrast CASZ1b is down regulated while CASZ1a is upregulated by agents that raise intracellular cAMP levels. CASZ1b and CASZ1a have no synergistic or antagonistic activities on the regulation of their target NGFR gene transcription. Specific restoration of CASZ1b in NB cells suppresses tumor growth *in vitro* and *in vivo*. Consistent with its function role, we find that low CASZ1b expression is significantly associated with decreased survival probability of neuroblastoma patients (p<0.02). This study indicates that although their mechanisms of regulation may be distinct, both CASZ1b and CASZ1a have largely redundant but critical roles in suppressing tumor cell growth.

## Introduction

CASZ1/Castor (*Cas*) gene was first characterized in drosophila where it encodes a zinc finger transcription factor that functions to regulate neural fate. Loss of *Cas* in Drosophila impairs differentiation and alters glial cell number and migration [1], [2], [3], [4]. In *Xenopus,* CASZ1 is required for heart development and the onset of cardiomyocytes differentiation at the ventral midline [5]. In humans, the CASZ1 gene localizes to chromosome 1p36, and deletion of this region frequently occurs in cancers such as neuroblastoma, melanoma, oligodendroglioma and breast cancers [6]. We have characterized two isoforms of CASZ1 gene from human, CASZ1a and CASZ1b [7]. CASZ1a, the full length isoform, comprises 1759 amino acids (AA) with 11 TFIIIA class C2H2 zinc finger; CASZ1b comprises 1166 AA that are identical to the first 1166 AA of CASZ1a but lacks the last 6 zinc fingers, so when described they were named hcasz11 and hcasz5, respectively [7]. A murine *in situ* hybridization study showed that total CASZ1 exhibits a dynamic expression pattern during neurogenesis [8], but the specific and quantitative expression of CASZ1a and CASZ1b isoforms has yet to be investigated. Recently we have shown that full length CASZ1 (CASZ1a isoform) functions as a neuroblastoma tumor suppressor [9], however a functional role for the CASZ1b has not been described to date.

The generation of multiple isoforms from a single gene, is believed to contribute to the complexity of higher organisms by enabling additional regulatory controls and insuring the accuracy of cellular signaling programs. Consequently, when studying the function of a given gene, it is imperative to delineate the functional potential of the corresponding protein products. For example, neuron-restrictive silencer factor (NRSF) silences neuronal genes expression, while its truncated variant sNRSF (or REST4) that lack the NRSF C-terminal region act as an activator rather than repressor of the transcriptome regulated by NRSF [10]. The tumor suppressor gene p73 has multiple isoforms with distinct functions with the transcriptionally active Tap73 isoform having a pro-apoptotic function and the ΔNp73 isoform functioning as a dominant negative, to attenuate TAP73 function and exerts anti-apoptotic functions [11]. However, there are also many cases in which the isoforms generated from a single gene play redundant roles. The p16γ is an alternate transcript of p16^INK4A^ and is co-expressed with p16^INK4A^ in cancer cells; p16γ functions indistinguishably from wild-type p16^INK4A^ with respect to CDK interactions and inhibition cell-cycle arrest and growth inhibition [12]. Two spliced forms of SEMA3F share common axonal chemorepulsive properties and are functionally redundant at regulating endothelial cell morphology *in vitro* [13]. We recently demonstrated that low total CASZ1 expression is associated with poor prognosis in neuroblastoma patients and the full length isoform CASZ1a suppresses neuroblastoma growth *in vitro* and *in vivo* [9], however it is important and necessary to assess the function of CASZ1b.

In *drosophila* and *Xenopus*, the predominant *Cas* isoform is homologous to CASZ1b. In human we have shown that CASZ1a and CASZ1b mRNA are the two major isoforms expressed in human cell lines as well as in adult human tissues such as heart and skeletal muscle by northern analysis [7]. A high degree of homology is found for both CASZ1a and CASZ1b protein in species like *Mus musculus*, *Rattus norvegicus* and *Canis familiaris*, and they share over 90% identity for either isoform (unpublished data). However, the nature of the functional relationship between CASZ1a and CASZ1b is not known. In this study, we find that CASZ1a and CASZ1b are dynamically expressed during neurogenesis and their expression is coordinately regulated during differentiation. CASZ1b has no synergistic or antagonistic activities to CASZ1a on gene transcription regulation. Primary neuroblastoma tumors that have either low CASZ1a or low CASZ1b have a poor outcome. Consistently, CASZ1b like CASZ1a is capable of suppressing neuroblastoma tumor growth.

## Results

### CASZ1a and CASZ1b are dynamically expressed during neurogenesis and are coordinately regulated *in vitro*

**Figure 1:**
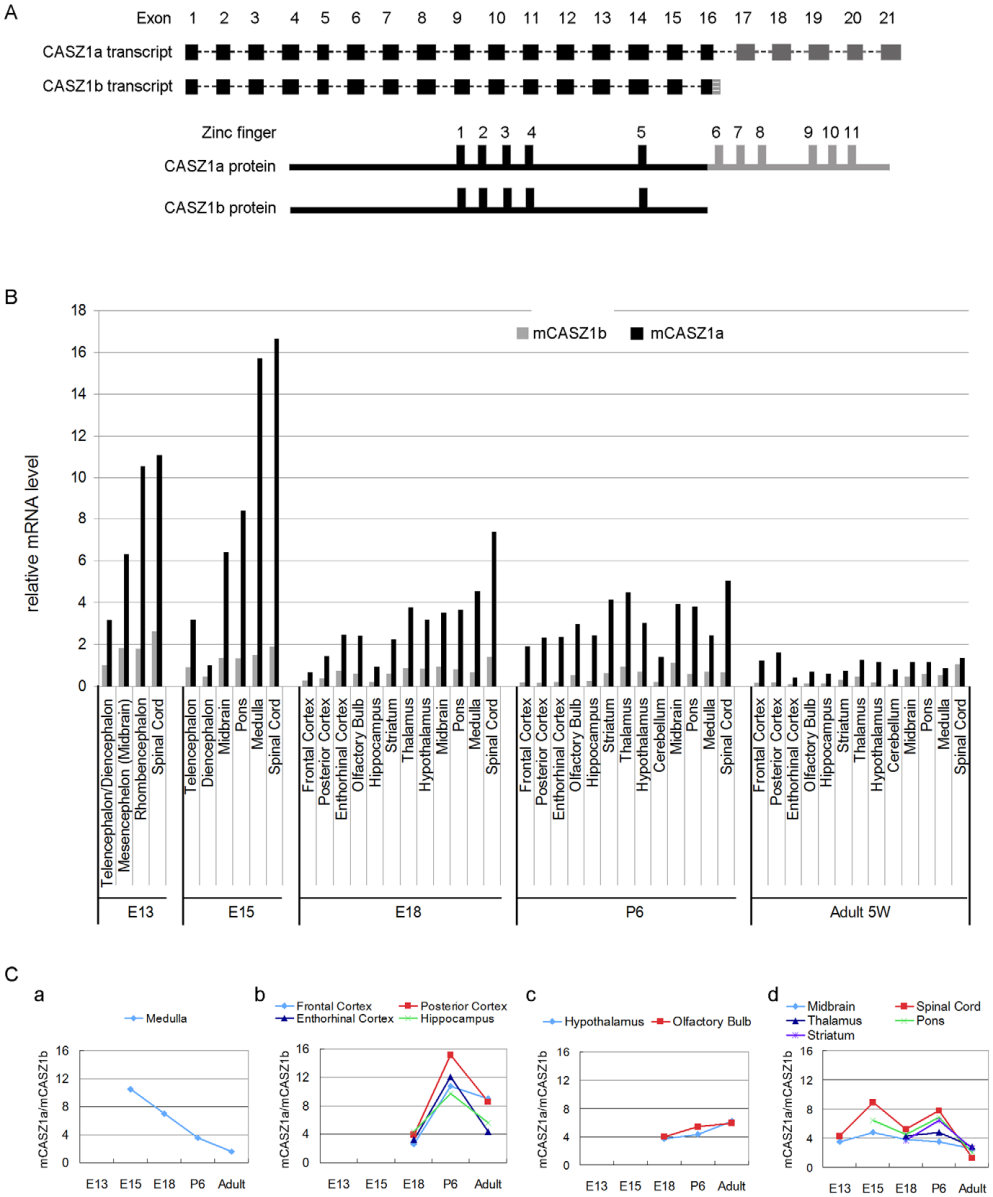
Mouse CASZ1a (mCASZ1a) and CASZ1b (mCASZ1b) co-exist during neurogenesis and exhibit different patterns of expression. A. CASZ1a and CASZ1b transcript structure (top). Exon 16 is differently spliced to produce these two transcripts; CASZ1a and CASZ1b protein structure (bottom); CASZ1b lacks the C-terminal region of CASZ1a, which includes 6 zinc fingers. B. mCASZ1a and mCASZ1b mRNA level in neuronal tissues were detected by real time PCR using mCASZ1a and mCASZ1b specific primers. The two isoforms are dynamically expressed in the nervous system during development with mCASZ1a having a relatively higher level of expression compared to mCASZ1b. C. The ratio of mCASZ1a and mCASZ1b mRNA varied during development in different regions of the brain: a. Medulla; b. Frontal, Posterior, Enthorithinal cortex and Hippocampus; c. Hypothalamus, Olfactory bulb; d. Midbrain, Spinal cord, Thalamus, Pons and Striatum.

CASZ1a and CASZ1b are encoded by the CASZ1 gene and generated by the use of two alternative polyadenylation sites and subsequent alternative splicing of exon 16. The CASZ1b protein lacks the last 6 zinc fingers and the C-terminus of the full length CASZ1a (Figure 1A). While total CASZ1 expression has been detected in the mouse nervous system by *in situ* hybridization [8], we quantitatively investigated the isoform specific expression of mouse CASZ1a (mCASZ1a) and CASZ1b (mCASZ1b) during neurogenesis, by designing primer pairs that target the mCASZ1a 3′-UTR and mCASZ1b 3′-UTR and performing real-time PCR (RT-PCR). The expression pattern of mCASZ1a and mCASZ1b in various neuronal tissues during neurogenesis is shown in Figure 1B. The mCASZ1a and mCASZ1b isoforms exhibit a dynamic expression pattern in all neuronal tissues evaluated including Telencephalon, Mesencephelon, Rhombencephalon, Midbrain, Pons, Frontal Cortex, Posterior Cortex, Enthorhinal Cortex, Olfactory Bulb, Hippocampus, Striatum, Thalamus, Hypothalamus, Cerebellum, Medulla and Spinal cord. The relative mCASZ1a and mCASZ1b expression levels were higher in the early stages (E13 and E15) and decreased in the later stage (adult). Both mCASZ1a and mCASZ1b isoforms are highly expressed in the medulla and spinal cord (Figure 1B). Peak levels of the mCASZ1a isoform were detected in the Spinal cord at E15 which was 1.5-fold higher than in E13, 2-fold higher than in E18 and 12-fold higher than in adult. mCASZ1b level in Spinal cord peaked at E13, it is 1.8-fold higher than in E18 and 3.7-fold higher than in adult (Figure 1B).

The mCASZ1a and mCASZ1b isoforms are co-expressed in neuronal tissues with a relatively higher level of mCASZ1a compared to mCASZ1b. The ratio of mCASZ1a to mCASZ1b (mCASZ1a/mCASZ1b) in different tissues varied from 1.3-fold (in adult Spinal cord) to 15-fold (in P6 posterior cortex) (Figure 1C). The ratio of mCASZ1a to mCASZ1b varied during development in different neuronal tissues. In the medulla, the mCASZ1a/mCASZ1b isoform ratio kept decreasing, from 10.5-fold in E15 to 1.6-fold in adult (Figure 1C, a). The mCASZ1a/mCASZ1b isoform ratio variation exhibits a similar pattern in some neuronal tissues during development and selected ones are shown in Figure 1C (Panels, b, c and d). These data suggest that the two isoforms might play a distinct role, work in concert to control the neuronal subtype specification and differentiation or be subject to different regulatory stimuli.

**Figure 2:**
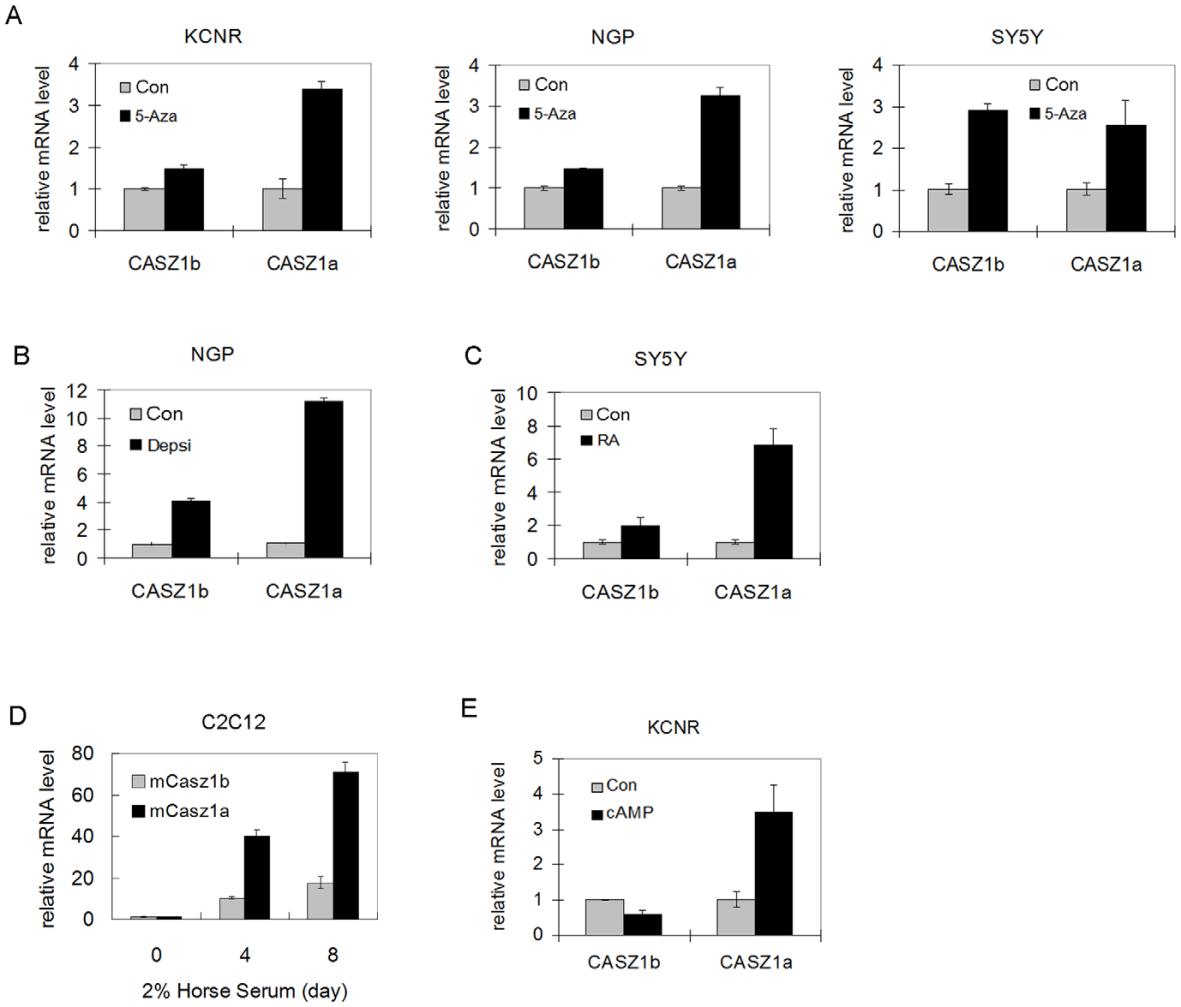
CASZ1a and CASZ1b are coordinately regulated. A. Neuroblastoma cell lines were treated with 2 µM demethylating agent 5-Aza-dC for 3 days. Both CASZ1a and CASZ1b mRNA levels were upregulated in KCNR, NGP and SY5Y as detected by RT-PCR using CASZ1a and CASZ1b specific primer (all p<0.02). B. NGP cells were treated with the class I histone deacetylase inhibitor, depsipeptide (5 ng/ml) for 24 hr. Both CASZ1a and CASZ1b mRNA levels were up-regulated by depsipeptide as detected by RT-PCR (all p<0.0002). C. Both CASZ1a and CASZ1b mRNA levels were up-regulated by 5 µM RA in SY5Y cells as detected by RT-PCR (all p<0.05). D. Both mCASZ1a and mCASZ1b mRNA levels were up-regulated when mouse C2C12 cells are cultured in 2% horse serum for 4 days or 8 days as detected by RT-PCR (all p<0.001). E. CASZ1b mRNA levels was down-regulated and CASZ1a mRNA levels was up-regulated by 2 µM dibutyryl cyclic AMP treatment for 48 hr in KCNR cells as detected by RT-PCR (all p<0.05).

The co-existence of mCASZ1a and mCASZ1 during neurogenesis suggests that they are coordinately regulated. To evaluate this we tested how epigenetic modulation or induction of differentiation affected CASZ1a and CASZ1b expression regulation in human neural crest derived neuroblastoma cells. After neuroblastoma cells were treated with the DNA demethylating agent 5-Aza-dC for 3 days, CASZ1a mRNA level was induced 2.2-3.5 fold in the NB cell lines tested while CASZ1b mRNA level was up-regulated 1.5-3 fold (Figure 2A). Treatment with the HDAC inhibitor depsipeptide (a Type I HDAC inhibitor) caused a 11-fold increase in CASZ1a mRNA level and 4-fold increase in CASZ1b mRNA level in NGP cells after 3 days (Figure 2B). Induction of SY5Y neuroblastoma cell differentiation with RA for 3 days was accompanied by 7-fold increase in CASZ1a mRNA level and 2-fold increase in CASZ1b mRNA level (Figure 2C). Using an alternative differentiation model we found that there was 71-fold increase in CASZ1a mRNA level and 18-fold increase in CASZ1b mRNA level in C2C12 myoblasts induced to differentiated into myotubes (Figure 2D). In contrast, we found 50% decrease in CASZ1b and 3.5-fold increase in CASZ1a mRNA level when KCNR cells were treated with agents that raise intracellular cAMP (Figure 2E).

### CASZ1a and CASZ1b are coexpressed and coordinately regulated at protein level

**Figure 3:**
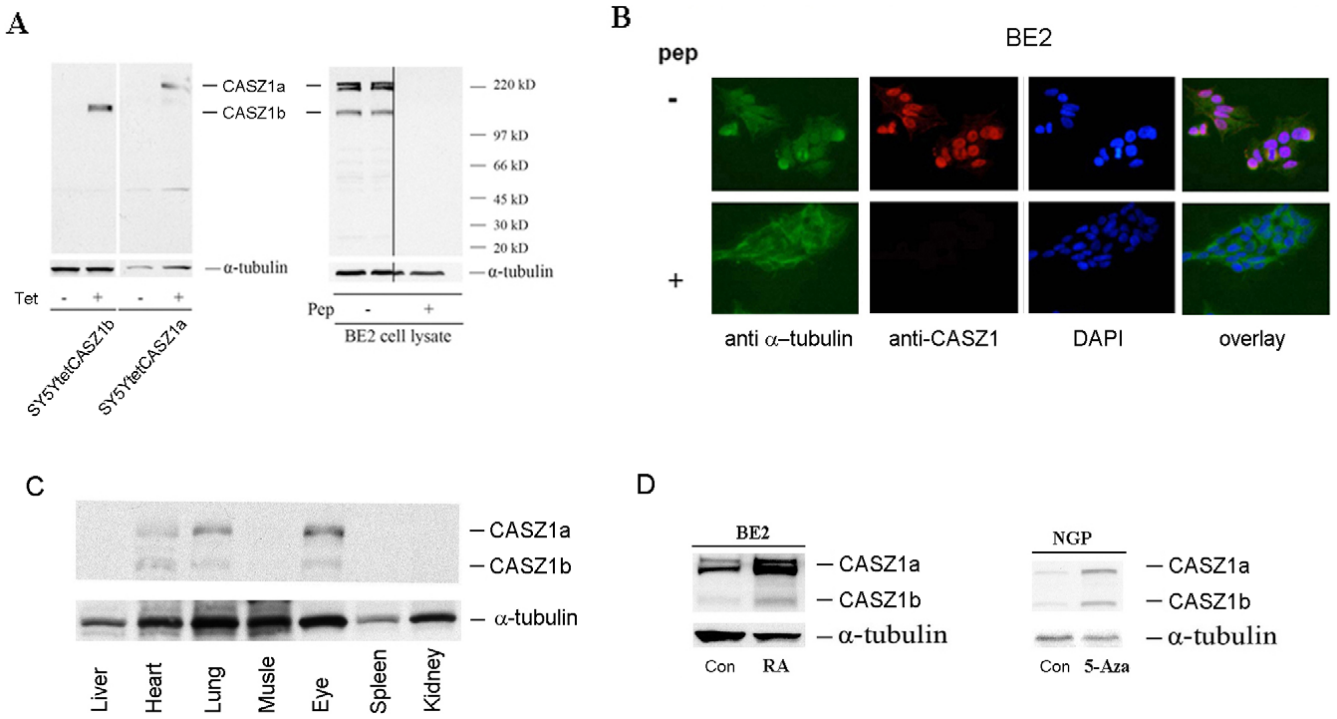
CASZ1a and CASZ1b are co-expressed and coordinately regulated at the protein level. A. Anti-CASZ1 antibody is specific to CASZ1a and CASZ1b. Induction of CASZ1a and CASZ1b expression in the SY5YtetCASZ1a and SY5YtetCASZ1b clones by Tet at 24 hours was visualized by immunoblotting the cell lysates with anti-CASZ1 antibody (left); endogenous CASZ1a and CASZ1b expression in neuroblastoma cells was visualized by immunoblotting the BE2 cell lysates with anti-CASZ1 antibody, and the specificity of the antibody was demonstrated by pre-incubation of immunizing peptide and loss of CASZ protein recognition with immunoblotting analysis (right). B. Endogenous CASZ1a and CASZ1b are predominantly expressed in the nucleus of neuroblastoma cells. The nuclear localization of CASZ1a and CASZ1b was characterized by the co-localization of Alex 568-labeled goat anti-mouse IgG and DAPI-stained nucleus of BE2 cells (top), and the specificity of the antibody was demonstrated by the blocking effect of antigen peptide on antibody recognition with immunoblotting analysis (bottom). C. The co-expression of murine CASZ1a and CASZ1b protein in heart, lung and eye of P6 mouse was visualized by immunoblot analysis of tissue lysates using an anti-CASZ1 antibody. D. The simultaneous up-regulation of CASZ1a and CASZ1b protein by either RA or 5-Aza-dC in neuroblastoma cell lines was visualized by immunoblot analysis of the cell lysates using the anti-CASZ1 antibody.

To assess CASZ1a and CASZ1b expression at the protein level, we utilized an affinity-purified anti-CASZ1 peptide antibody, which recognizes an epitope (AA 923-936) shared by both CASZ1a and CASZ1b. Our first level of evaluation was to test whether the antibody recognizes the exogenously expressed CASZ1a and CASZ1b proteins. FLAG-tagged CASZ1a and CASZ1b in Tet-on vector was stably transfected into SY5Y neuroblastoma cell lines that express Tet repressor (SY5YtetCASZ1a cells or SY5YtetCASZ1b cells). Whole protein lysates from SY5YtetCASZ1a or SY5YtetCASZ1b clones cultured in the absence or presence of 1ug/ml tetracycline (TET) were separated on a 4–12% SDS-page gel and transferred to a nitrocellulose membrane. Induction of CASZ1a and CASZ1b expression by TET was visualized by immunoblotting whole cell lysate with the affinity-purified rabbit anti-CASZ1 antibody; however, no CASZ1a or CASZ1b band was seen in control lanes (non-TET treated) (Figure 3A, left). Endogenous CASZ1a and CASZ1b expression in BE2 cell line were detected using the anti-CASZ1 antibody; and the specificity of the anti-CASZ1 antibody was demonstrated by pre-incubation of the antibody with immunizing peptide followed by immunoblotting analysis (Figure 3A, right). We saw two bands at CASZ1a's relative molecular weight of 220KD in BE2 cell lysates. The extra band might be a post-translation modification (such as phosphorylation) of CASZ1a protein. Endogenous CASZ1a and CASZ1b are also predominantly expressed in the nucleus (Figure 3B, upper panel) consistent with our findings for transfected CASZ1 [7]. Prior incubation of the anti-CASZ1 antibody with the immunizing peptide blocked CASZ1 staining demonstrated the specificity of this staining (Figure 3B bottom panel).

To determine the CASZ1 gene expression pattern in normal tissues, whole protein lysates from P6 mouse tissues were separated by on 4–12% SDS-page gel and western blot analysis was performed using anti-CASZ1 antibody. We found that mouse CASZ1a and CASZ1b proteins are co-expressed in heart, lung and eye (Figure 3C). Moreover both isoforms are coordinately up-regulated about 2-fold by RA treatment of BE2 cells or almost 3-fold by 5-Aza-dC treatment of NGP cells (Figure 3D). These data demonstrate that CASZ1a and CASZ1b are co-expressed and coordinately regulated at both the mRNA and protein levels.

### CASZ1b is neither a dominant negative nor a synergistic factor of CASZ1a at regulating gene expression

**Figure 4:**
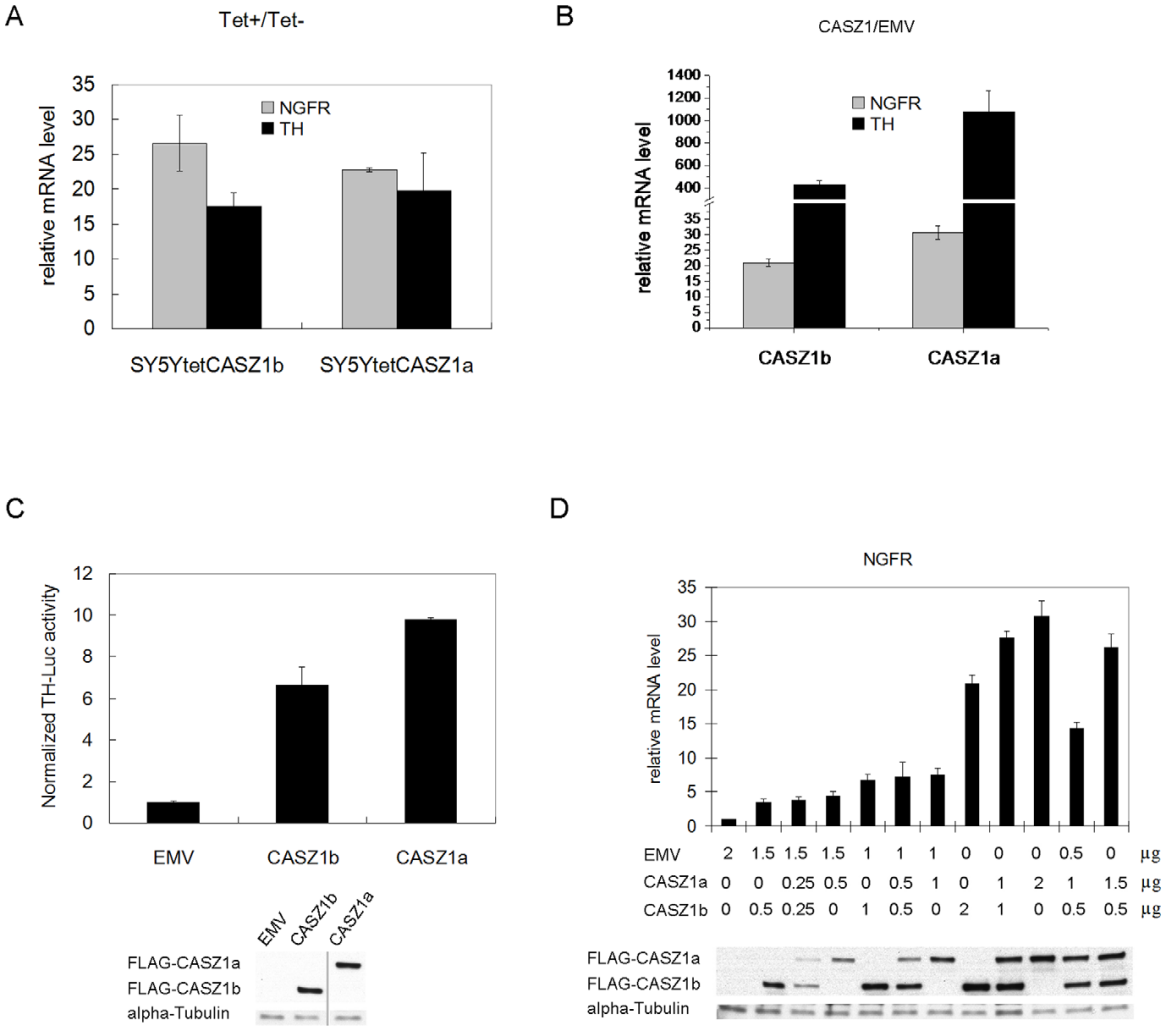
There is no antagonist or synergistic effect between CASZ1b and CASZ1a at the level of gene transcription. A. Induction of NGFR and TH expression in the SY5YtetCASZ1a and SY5YtetCASZ1b clones after induction of CASZ1a and CASZ1b by Tet at 24 hours was confirmed by real time PCR (all p<0.01). B. pCMVTag2A empty vector (EMV) or pCMVTag2A-CASZ1a (pCMVtag2A-CASZ1b) was transiently transfected into 293T cells, and the fold induction of NGFR and TH by CASZ1a or CASZ1b compared to EMV at 24 hr was detected by real time PCR (all p<0.01). C. TH-Luciferase construct was activated by CASZ1b and CASZ1a 24 hours after transfection of HEK293T cells at 24 hr (p<0.0005). D. The degree of NGFR induction by co-transfection of CASZ1b plus CASZ1a into HEK293T cells is similar to the cells that were transfected by single isoform.

CASZ1b and CASZ1a induce NGFR and Tyrosine Hydroxylase (TH) in SY5Y cells as well as HEK293T cells (Fig 4A, B) upon transfection of either CASZ1b or CASZ1a. Moreover, similar results are found for activation of the TH promoter in HEK293T cells (Figure 4C). Thus both isoforms are functionally equivalent in inducing TH and NGFR. This suggests that the short isoform CASZ1b is not a dominant negative of the longer CASZ1a isoform. To more formally assess the synergistic or antagonistic activities of the CASZ1 isoforms we co-transfected CASZ1a and CASZ1b plasmids into HEK293T cells and assessed the expression of NGFR by RT- PCR. NGFR mRNA expression was induced by CASZ1a, and increasing the amount of the transfected plasmid (from 0.5 µg to 2 µg) resulted in a stronger induction (4-fold to 30-fold) (Figure 4D). The fold induction of NGFR by CASZ1a plus CASZ1b was similar to the fold induction of NGFR by a single isoform (or the average of the two isoforms) when controlling for the total amount of plasmid. This occurred even when different ratios of CASZ1a and CASZ1b isoform plasmids were transfected (Figure 4D). This result suggests that CASZ1a and CASZ1b have neither antagonistic nor synergistic activities on NGFR gene transcription regulation.

### Either low CASZ1a or CASZ1b is associated with poor prognosis in Neuroblastoma

**Figure 5:**
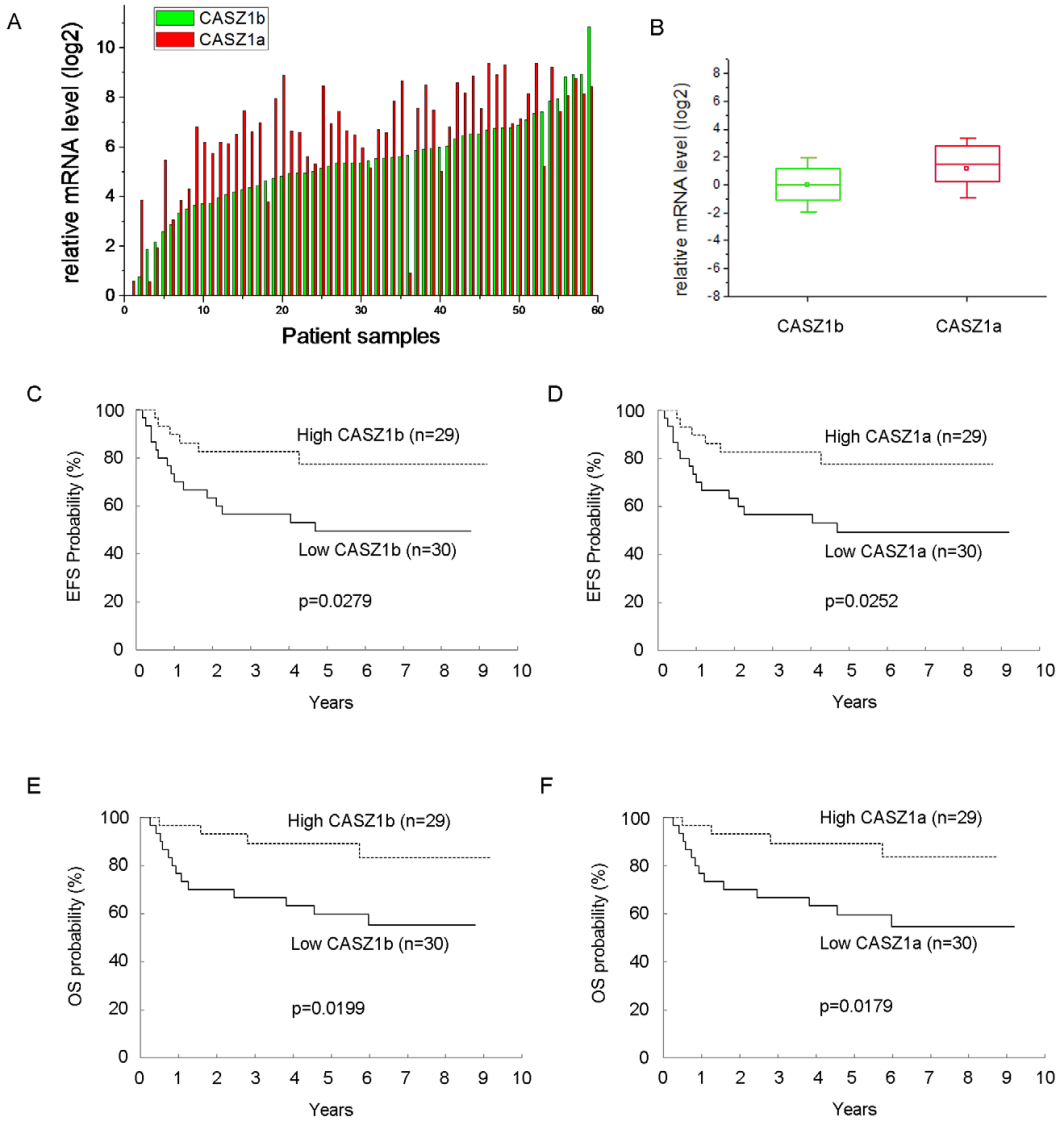
Loss of either CASZ1b or CASZ1a expression is correlated with poor prognosis in neuroblastoma. A. CASZ1a and CASZ1b have similar expression patterns in primary neuroblastoma samples. B. The average of CASZ1a mRNA level is about 2-fold higher than CASZ1b (p<0.002). C. Kaplan-Meier curves of EFS for low (<0.9468) vs. high (≥0.9468) CASZ1b expression in 59 neuroblastoma patients. D. Kaplan-Meier curves of EFS for low (<0.9195) vs. high (≥0.9195) CASZ1a expression in 59 neuroblastoma patients. E. Kaplan-Meier curves of OS for low (<0.9468) vs. high (≥0.9468) CASZ1b expression in 59 neuroblastoma patients. F. Kaplan-Meier curves of OS for low (<0.9195) vs. high (≥0.9195) CASZ1a expression in 59 neuroblastoma patients (all p<0.05).

Our previous RT-PCR analysis showed that low expression of total CASZ1 is significantly correlated with decreased survival probability [9]. However, since the ratios of the two isoforms are often different under different conditions we sought to evaluate their expression pattern in primary tumors from neuroblastoma patients. We evaluated CASZ1a and CASZ1b mRNA expression in 59 primary neuroblastoma patients' tumors by real-time PCR using CASZ1a and CASZ1b specific primers. We found that both CASZ1a and CASZ1b mRNA are detected in all of the tested primary neuroblastoma tumors. The relative level of CASZ1a was higher than CASZ1b in the majority of the samples (Figure 5A) and the mean steady-state level of CASZ1a was approximately 2-fold higher than CASZ1b (Figure 5B).

The median values of CASZ1b (0.9468) and CASZ1a (0.9195) expression were used to dichotomize the patients into two groups (< median vs. ≥ median). Tests of association of dichotomized CASZ1b and CASZ1a with age, International Neuroblastoma Staging System (INSS) stage, risk, *MYCN* status, Shimada histology, ploidy, 1p loss of heterozygosity (LOH), and 11q LOH were performed (Table 1, 2). Statistically significant associations were found between all factors except ploidy for CASZ1b (Table 1), and except age and ploidy for CASZ1a (Table 2). In all the statistically significant cases, high expression of CASZ1 isoforms was associated with the more beneficial level of the factor (<18 months, not stage 4, low or intermediate risk, non-amplified MYCN, favorable Shimada histology, no 1p LOH) except with 11q, which was reversed. The patients with high CASZ1b had a statistically significantly better EFS (p = 0.0279) and OS (P = 0.0199) than that for patients with low CASZ1b expression (Figure 5C, E). Similarly, patients with high CASZ1a expression had statistically significantly better EFS (p = 0.0252) and OS (p = 0.0179) than that for patients with low hCASZ11 expression (Figure 5D, F).

**Table 1:** Association of CASZ1b and CASZ1a Expression Level with Prognostic Factors (n = 59).

Factor	n	Number (%) of Patients with CASZ1b Expression ≥ 0.9468 (median)	p-value*
Age			
<18 months	33	22 (67)	0.0038
≥18 months	26	7 (27)	
INSS Stage			
1,2,3,4s	32	20 (63)	0.0370
4	27	9 (33)	
Risk Group			
Low/Intermediate	30	21 (70)	0.0017
High	29	8 (28)	
*MYCN*			
Not amplified	39	26 (67)	0.0003
Amplified	20	3 (15)	
Shimada histology			
Favorable	27	21 (78)	0.0002
Unfavorable	29	8 (28)	
Unknown	3		
Ploidy			
Hyperdiploid	38	22 (58)	0.1033
Diploid	21	7 (33)	
1p			
No LOH	39	25 (64)	0.0030
LOH	17	3 (18)	
Unknown	3		
11q			
No LOH	39	15 (38)	0.0186
LOH	17	13 (76)	
Unknown	3		

*From 2-sided Fisher's Exact Test.

**Table 2:** Association of CASZ1b and CASZ1a Expression Level with Prognostic Factors (n = 59).

Factor	n	Number (%) of Patients with CASZ1a Expression ≥0.9195 (median)	p-value*
Age			
<18 months	33	20 (61)	0.0673
≥18 months	26	9 (35)	
INSS Stage			
1,2,3,4s	32	20 (63)	0.0370
4	27	9 (33)	
Risk Group			
Low/Intermediate	30	21 (70)	0.0017
High	29	8 (28)	
*MYCN*			
* *Not amplified	39	26 (67)	0.0003
Amplified	20	3 (15)	
Shimada histology			
Favorable	27	18 (67)	0.0372
Unfavorable	29	11 (38)	
Unknown	3		
Ploidy			
Hyperdiploid	38	22 (58)	0.1033
Diploid	21	7 (33)	
1p			
No LOH	39	25 (64)	0.0083
LOH	17	4 (24)	
Unknown	3		
11q			
No LOH	39	14 (36)	0.0030
LOH	17	14 (82)	
Unknown	3		

*From 2-sided Fisher's Exact Test.

### Like CASZ1a, CASZ1b suppresses neuroblastoma cell proliferation

**Figure 6:**
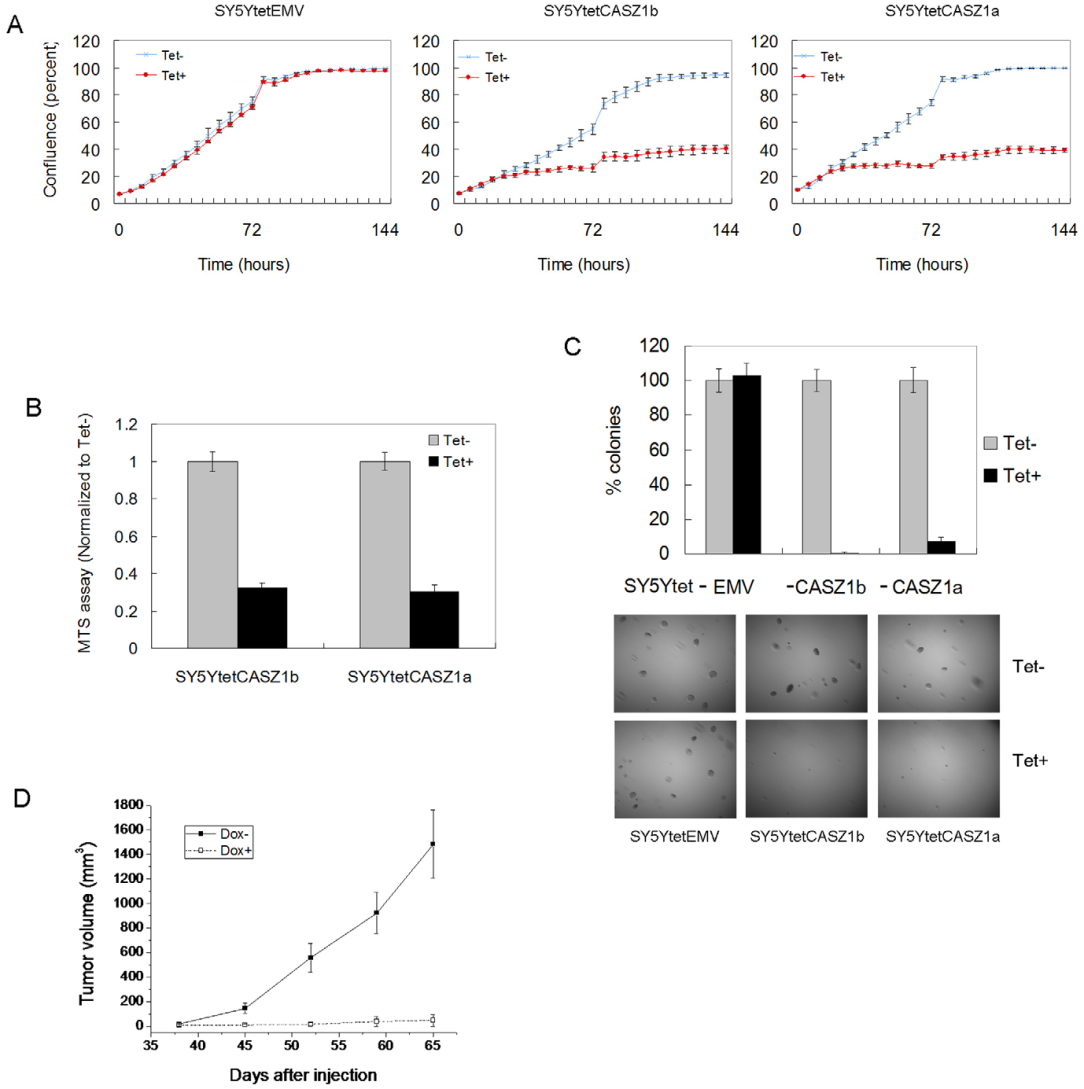
Like CASZ1a, CASZ1b suppresses neuroblastoma cell growth. A. Induction of CASZ1b by Tet in SY5YtetCASZ1b cells inhibited cell growth, and the magnitude of inhibition was similar to CASZ1a in SY5YtetCASZ1a cells. The cell confluence was measured at different time point using IncuCyte instrument (P<0.05 for all time points since day 2). B. Similar to CASZ1a, the induction of CASZ1b by Tet inhibited proliferation of SY5YtetCASZ1b cells as assessed using a MTS assay on day 7 (p<0.005). C. Similar to CASZ1a, the induction of CASZ1b by Tet decreased clonogenicity of SY5YtetCASZ1b cells in soft agar (p<0.005). D. SY5YtetCASZ1b cells were subcutaneously injected into nude mice. The induction of CASZ1b expression significantly inhibited tumor growth in the xenograft model (n = 5 mice per group, total 10 mice; Volume = long × short^2^/4; data are shown as mean ± SEM, all time point p<0.02 since day 45).

The similarities in transcriptional activity by the CASZ1 isoforms suggest that these isoforms may also have similar functions. Previously we found that CASZ1a suppresses neuroblastoma cell proliferation [9] and in this study, we found that upon CASZ1b induction, neuroblastoma cell proliferation was inhibited ∼80% in SY5Y cells by either a cell confluence assay or MTS assay. Growth of the empty vector control cell lines was not affected by Tet treatment (Figure 6A, B).

To test whether CASZ1b inhibits tumorigenicity of neuroblastoma cells, anchorage-independent growth was assessed by soft agar clonogenicity and visible clones were counted after 4 weeks. Similar to CASZ1a clones, the CASZ1b expressing cells showed an over 90% decrease in soft agar clonogenicity compared to the controls (Figure 6C). To assess *in vivo* tumorigenicity, SY5YtetCASZ1b cells were implanted at a subcutaneous site into mice that had received normal food or doxycycline containing food during the prior week (5 mice per group). Doxycycline-treated animals showed decreases in tumor growth. An inhibition of tumor growth of 90% was detected at the 9^th^ week compared to placebo-treated mice (Figure 6D).

## Discussion

The generation of multiple isoforms from a single gene ensures the accuracy of cellular signaling programs and enables additional regulatory controls. We describe here the characterization of an evolutionarily conserver, short alternatively spliced isoform of the CASZ1 gene, CASZ1b. CASZ1b isoform lacks 1/3 of the C-terminal region of the full length CASZ1a isoform. Expression of CASZ1b is detected in all CASZ1a-expressing neuroblastoma samples and mouse tissues. CASZ1a and CASZ1b are coordinately regulated when neuroblast and myoblast cells are induced to differentiate, but the two CASZ1 isoforms are characterized *in vivo* by a temporal and regional specific regulation during neurogenesis. Functionally, CASZ1b behaves similar to CASZ1a at regulating gene expression and the co-expression of CASZ1b and CASZ1a shows neither cross antagonistic or synergistic activities. Moreover low CASZ1b or CASZ1a expression is associated with poor prognosis of neuroblastoma patients and the restoration of CASZ1b expression in neuroblastoma cells suppressing neuroblastoma cells growth *in vitro* and *in vivo*.

CASZ1 in drosophila is a neuronal fate-determination gene and controls the nervous system development [1], [2], [4]. There are highly evolutionarily conserved DNA elements in the noncoding region of CASZ1 gene from drosophila to human [14], [15], [16]. The highly conserved noncoding elements are strongly associated with developmental regulatory genes [16], [17] suggesting that CASZ1 must be playing some fundamental role in mammalian nervous system development as well. In this study, we find that during neurogenesis, mouse CASZ1 (mCASZ1a) and CASZ1b (mCASZ1b) exhibits a dynamic expression pattern. Both mCASZ1a and mCASZ1b are expressed at a relatively higher levels during early development (E13, E15) compared to a later stages or in adults (P6, adult), and the levels vary in different neuronal subtypes as well (Figure 1B). The spatial and temporal expression patterns of mCASZ1a and mCASZ1b during neurogenesis suggest an important role in the control of neuronal subtype specification, differentiation and neuronal migration. Although mCASZ1a and mCASZ1b are co-expressed in all the tested neuronal tissues, their ratios vary at spatially and temporally. (Figure 1C). It is possible that there is a modulating role between mCASZ1b and mCASZ1a in the control of nervous system development, and this modulating role is dependent on the ratio between these two isoforms.

The co-existence of CASZ1a and CASZ1b suggests the two isoforms are coordinately regulated. In the *in vitro* models we find that the two isoforms are simultaneously upregulated when neuroblastoma cells or myoblast cells are induced to differentiate by RA or 2% horse serum (Figure 2 A–D), which is consistent with the bioinformatic assay that CASZ1a and CASZ1b share the same promoter. We find that the induction of CASZ1a at mRNA level is greater than the induction of CASZ1b in the *in vitro* model and CASZ1a mRNA level is higher than CASZ1b in the mouse neuronal tissues, suggesting CASZ1a and CASZ1b mRNA have different stability or there is a different post-transcriptional regulation. microRNA (miRNA) are post-transcriptional regulators that target 3′UTR to control gene expression by degrading or repressing target mRNAs [18]. Using an online miRNA search tool (http://www.microrna.org/microrna/getGeneForm.do), we find that there are 26 conserved miRNAs with good mirSVR scores that target CASZ1a mRNA 3′UTR (NM_001079843). There are 26 unique miRNAs target CASZ1b mRNA 3′UTR (NM_017766), which are different from those miRNAs that target CASZ1a (unpublished data). So CASZ1a or CASZ1b mRNA levels may be regulated by the tissue and cell type specific expression of miRNAs. Agents that raise intracellular cAMP decrease CASZ1b and increase CASZ1a mRNA levels (Figure 2E) suggesting that the cAMP pathway might regulate CASZ1 mRNA stability through miRNAs. Consistent with CASZ1 expression pattern, miRNA plays a central role to control neurogenesis and tumorigenesis [18], [19]. A future study will focus on identifying bona-fide miRNAs targets to CASZ1a and CASZ1b to fully understand the regulation of their expression during neurogenesis.

Frequently different isoforms from a single gene have distinct functions. For example, the full length TAp73 and the ΔNp73 isoforms are transcribed from the p73 gene. The ΔNp73 isoform acts as a dominant-negative inhibitor of TAp73 to prevent target gene induction by TAp73 [11]. Co-transfection of CASZ1a with CASZ1b into HEK293T cells have the same effect as transfection of CASZ1a alone on the target NGFR gene expression (Figure 4) indicating that there is no synergistic or antagonistic effect on a target gene between CASZ1a and CASZ1b. In the transient transfection experiment, overexpression CASZ1a in HEK293T cells induces more NGFR and TH than CASZ1b does (Figure 4B, C), suggesting that CASZ1a may enable more active transcription from a target gene. This may be because the extra 6 zinc fingers of CASZ1a stabilize the DNA binding or recruit more co-activators to enhance gene transcription. CASZ1a is a neuroblastoma tumor suppressor gene that suppresses tumor growth *in vitro* and *in vivo* [9]. Upon restoration of CASZ1b expression in neuroblastoma cells we find that like CASZ1a, CASZ1b suppresses tumor growth (Figure 6). Consistently, we find low CASZ1a or CASZ1b are associated with poor prognosis of neuroblastoma patients (Figure 5), which is similar to what we find for total CASZ1 [9].

There is evidence that isoforms generated from a single gene may play redundant roles. The p16γ is an alternate transcript of p16^INK4A^ and co-expresses with p16^INK4A^ in cancer cells; p16γ functions similarly to wild-type p16^INK4A^ at inhibiting cell growth [12]. Two spliced forms of SEMA3F are functionally redundant at regulating endothelial cell morphology *in vitro* although they are differentially expressed in nervous system [13]. Another more commonly recognized example is Wilm's tumor gene, WT1, which encodes up to 24 different isoforms which have distinct but also overlapping cellular and developmental functions [20]. Our results suggest CASZ1a and CASZ1b function similarly at regulating gene transcription and suppressing tumor growth. These findings provide fundamental information for the future study of the association of CASZ1 gene encoded proteins with pathogenesis.

In the context of gene transcription regulation and tumor growth, we found that the CASZ1a and CASZ1b isoforms play redundant roles. It is possible that either isoform has the capacity to compensate for each other's loss during normal development; when one isoform is accidently degraded or silenced; the other isoform may undertake the role. We believe that this redundant expression and largely overlapping functions of CASZ1a and CASZ1b isoforms indicate that expression of the CASZ1 gene is an important regulator of normal development. In fact, it has been proposed that redundancy as a conserved design of genetic networks [21]. While we find similarities in function this is limited by our knowledge of CASZ1's known functions as delineated in *in vitro* studies. It is possible that they have distinct yet unknown functions. CASZ1b lacks the C-terminal region of CASZ1a, which containing 6 zinc fingers, this may result in changes in the three-dimensional differences for the two isoforms. The unique six zinc fingers of CASZ1a might contribute its specific protein-DNA binding or protein-protein interaction role; and the potential differential post-translation modifications of CASZ1a and CASZ1b isoforms such as phosphorylation or ubiquitination may also contribute to their distinct role in a different context. Further investigation to pursue these possibilities may be warranted.

In summary, we have reported the functional characterization of the short isoform of CASZ1 gene, CASZ1b. CASZ1b functions similar to the full length isoform CASZ1a at regulating gene expression, suppressing neuroblastoma cell growth *in vitro* and *in vivo*. CASZ1b does not play synergistic or antagonistic role to CASZ1a based on gene transcription regulation. We conclude that the evolutionarily conserved CASZ1b isofom also functions as a candidate tumor suppressor. The neuronal tissue-specific patterns of expression and regulation of CASZ1a and CASZ1b suggest the two isoforms may play a fundamentally similar role but at different times and in different cell types. Future studies of CASZ1a or CASZ1b specific knock out or transgenic mouse will provide further insight into our understanding of the relationship between CASZ1a and CASZ1b and their importance during neurogenesis and how low levels impact tumorigenesis.

## Materials and Methods

### Cell culture

Human neuroblastoma cell lines SK-N-BE2 (BE2), SMS-KCNR, NGP and SH-SY5Y (SY5Y) were cultured in RPMI 1640 supplemented with 10% fetal bovine serum, 2 mM L-glutamine, 100 µnit/ml penicillin and 100 µg/ml streptomycin as described previously [7], [9]. Mouse C2C12 myoblasts were maintained in DMEM supplemented with 10% fetal bovine serum, 50 U/ml penicillian, and 50 µg/ml streptomycin [7]. To induce C2C12 cell differentiation, the cells were plated into 6-well plate, 24 hr later when cells were 80% confluent, the medium was switched to the differentiation medium containing DMEM and 2% horse serum, which was changed every other day. Human HEK293T cells were obtained from ATCC company (Manassas, VA, USA), and the cells were cultured in Dulbecco's modified Eagle's media, supplemented with 10% fetal bovine serum, 2 mM L-glutamine, 100 µnit/ml penicillin and 100 µg/ml streptomycin.

### Stable clones

Full-length FLAG-tagged CASZ1 (CASZ1a) cDNA and FLAG-tagged short isoform CASZ1 (CASZ1b) were cloned into the tetracycline (Tet) inducible vector pT-REx-DEST30. The control vector pDest-30 with an out-of-frame CAT gene with no start codon served as the empty vector control.

Tetracycline (Tet)-inducible expression is dependent on expression of the Tet repressor in the recipient cells. Recipient cells have been prepared by stable transfection of the pcDNA6/TR (Tet repressor) plasmid (Invitrogen) into SY5Ytet12 cells [22] that are selected with blasticidin. CASZ1a or CASZ1b in pT-REx-DEST30 or an empty vector control were transfected into SY5Ytet with G418 (300–500 µg/ml) and selected with blasticidin (5 µg/ml). Antibiotic-resistant transfectants were isolated and evaluated for tetracycline (Tet) (1 µg/ml) regulated CASZ1a or CASZ1b expression. Stable clones expressing CASZ1a are labeled SY5YtetCASZ1a, and stable clones expressing CASZ1b are labeled SY5YtetCASZ1b. The stable clones with empty vector are labeled SY5Ytetemv.

### Western Blot and Indirect Immunofluorescence Assay

Protein extraction, western blot analysis and indirect immunofluorescence assay were performed as described previously [7]. CASZ1 antibody (Rockland Immunochemical for Research) recognizes both CASZ1a and CASZ1b isoforms. The affinity purified rabbit polyclonal antibody was raised against a peptide mapping with an internal region of CASZ1a (NP_001073312.1, amino acid 923-936, sequence: HEASQDRSLDLTVK) of human origin, and the peptide sequence is 100% conserved in mouse. For Western Blot analyses, an anti–Flag (1∶1000) antibody from Sigma, antibodies to endogenous CASZ1 (1∶2000) from Rockland, α-tubulin (1∶2000) from Santa Cruz were used to detect each protein expression. For indirect immunofluorescent cell staining, cells were incubated with anti- CASZ1 (1∶500) or α-Tubulin (1∶500) (Molecular Probes) for 1 hr at room temperature, washed 3 times, followed by incubation with goat anti-rabbit IgG-Alex 568 and goat anti-mouse IgG-Alex 488 (1∶300) (Molecular Probes) for 45 min at room temperature.

### Real-time quantitative PCR

The primer sets used for the realtime PCR were shown in Table 3. Quantitative real-time PCR was performed on ABI Prism 7000 (PE Applied Biosystems) using SYBR Green SuperMix as described previously and β-Actin was used as normalization control [7]. The PCR was performed in duplicate or triplicate and repeated at least once. For the primary tumor study, total RNA from neuroblastoma tumors from 59 patients prior to chemotherapy was provided by the Children's Oncology Group (COG) Neuroblastoma Biology Group Committee. RNA (2 µg) was used as template for cDNA synthesis using Superscript III (Invitrogen), and CASZ1a and CASZ1b levels assessed by SYBR realtime PCR using CASZ1a and CASZ1b specific primer sets. PCR reactions were performed according to the manufacture's protocol. For mouse CASZ1a and CASZ1b tissue distribution studies, tissuescan mouse developmental qPCR array was used, which contains fist strand DNA from 48 tissues at five stages and normalized to GAPDH (OriGene Technologies).

**Table 3:** List of Primers Used for real-time PCR.

**Gene**	**Forward Primer**	**Reverse Primer**
β-Actin	GCCAACCGCGAGAAGATGA	CATCACGATGCCAGTGGTA
CASZ1	CAAAACAGACTCCATCACCACG	GTGCTGGCTGCCCGAGAAC
CASZ1a	GGATGCTGAGACAGATGAGTGC	CTGTCGGCATAGAGATGGTGTT
CASZ1b	TCCCTCCGAGCCTCCGTAT	GGGTCCCTTCCACCCAAGA
mβ-Actin*	TAAGGCCAACCGTGAAAAGAT	ACCAGAGGCATACAGGGACAG
mCASZ1*	GCAGAAGAGCCCTCAAAAGATAA	GAAGCAGCGTAGTCCCTCAGA
mCASZ1a*	AGGCAATGAGGCCACTTAGA	GCACTAGCCAGAACCTGGAG
mCASZ1b*	CCCAGGCTGAGTCAATAGGA	TGAGAACAGGAAGCCACACA
NGFR	ACCTCATCCCTGTCTATTGCTCC	GCTGTTGGCTCCTTGCTTGTT
TH	CCTACCAAGACCAGACGTACCAGTCA	TGCACCTAGCCAATGGCACTCA

*: These primers are for mouse genes. All the other primers are for human genes.

### Promoter activity assay

HEK293T cells were used for promoter activity assay. Cells in 24 – well plate were transfected using lipofectamine 2000 (Invitrogen) following the manufacturer's protocol. Tyrosine hydroxylase promoter – pGL4.1-luc construct (TH-luc) is generously provided by Gregory Wray, which contains 2kb of TH promoter [23]. A CMV-driven β-galactosidase construct was co-transfected with TH-luc and pCMV-FLAG_CASZ1a or pCMV-FLAG_CASZ1b in order to provide an internal control for transfection efficiency. Luciferase activity was quantified after 24 hours using the Dual Luciferase Reporter Assay System (Promega), and beta-galactosidase activity was measured concomitantly with the Luminescent Beta-galactosidase Detection Kit II (Clontech). The experiments were repeated for three times.

### Cell proliferation and clonogenicity assays

To assess the effect of CASZ1 on neuroblastoma cell proliferation, SY5YtetCASZ1a and SY5YtetCASZ1b cells were plated into 24-well plate. The next day, Tet was added to the RPMI-1640 containing 10% fetal calf serum. Cells were cultured in the IncuCyte (ESSEN INSTRUMENTS) and cell confluence was measured every 6 hr for 6 days using the IncuCyte software. MTS assay was also applied to assess cell proliferation for both SY5Y and AS cells that expressing CASZ1a or CASZ1b. To assess effects of CASZ1a or CASZ1b on anchorage independent cell growth, 1×10^4^ cells were cultured in 0.7% top agarose in media containing G418 and Blasticidin (± Tet) plated on a layer of 1.4% bottom agar/RPMI to prevent the adhesion of cells to the culture plates. Medium was changed 3x/week and visible colonies counted after 4 weeks.

### Animals

The animal studies were approved by the Animal Care and Use Committee of the National Cancer Institute, and all animal treatments, including their housing, were in accordance with institutional guidelines (PB-023).

### *In vivo* tumorigenesis

Suspensions of SY5YtetCASZ1b (2×10^6^ cells were mixed with an equal volume of Matrigel solution (Trevigen) and implanted subcutaneously in the dorsal flank of 10 SCID mice (6- to 8-week-old female mice) per cell line. Control mice received regular food, and the other half received doxycycline containing food for 1 week prior to tumor implantation and during the course of the experiment. The tumors were measured once a week, and sacrificed once the tumor diameter reached 20 mm.

### Statistical analyses

Patients were categorized as having low or high levels of CASZ1b and CASZ1a expression based on whether their values fell below or above the median. Stratification with regard to age (<18 months vs. ≥18 months), INSS stage (not stage 4 vs. stage 4), risk group (low & intermediate vs. high), *MYCN* status (not amplified vs. amplified), Shimada histology (favorable vs. unfavorable), ploidy (hyperdiploid vs. diploid), 1p LOH (no LOH vs. LOH), and 11q LOH (no LOH vs. LOH) was examined. The association of CASZ1b and CASZ1a expression levels with these prognostic factors was examined via a two-sided Fisher's exact test.

The method of Kaplan and Meier [24] was used to generate survival curves, with standard errors per Peto et al. [25]. For EFS, an event was defined as relapse, progression, secondary malignancy, or death from any cause. For overall survival (OS), death was the only event considered. The log-rank test was used to test for differences between survival curves.

Additional statistical analyses of continuous data were performed using a *t*-test. Values in the graphs are expressed as means ± SEM or SD. The statistical tests were two-sided. P-values less than 0.05 were considered statistically significant.
